# 

**Published:** 2023-09

**Authors:** Peter Raspor, Sonja Smole Možina, Irena Vovk

**Affiliations:** 1 Emeritus Professor of Microbiology and Biotechnology, University of Ljubljana, Jamnikarjeva 101, 1000 Ljubljana, Slovenia; 2 Chair of Biotechnology, Microbiology and Food Safety, Department of Food Science and Technology, Biotechnical Faculty, University of Ljubjana, Jamnikarjeva 101, 1000 Ljubljana, Slovenia; 3 Laboratory for Food Chemistry, Department of Analytical Chemistry, National Institute of Chemistry, Hajdrihova 19, 1000 Ljubljana, Slovenia

This special issue of Food Technology and Biotechnology contains selected papers presented at the 11^th^ Central European Congress on Food and Nutrition (CEFood 2022) entitled ’Food, technology and nutrition for healthy people in a healthy environment’, which was held from 27 to 30 September 2022 in Čatež ob Savi (Slovenia). Since 2002, CEFood congresses have been held biannually in different CEI countries, the 11^th^ was held in Slovenia again after 20 years. The scientific and professional framework of the 1^st^ CEFood congress in 2002 already provided a solid basis and guarantee for the CEFood congresses. In 2006, the European Federation of Food Science and Technology (EFFoST) expressed a clear wish to join this network, due to the good and efficient organisation based on the commitment of the main organisers of CEFood congresses and their teams. Above all, respect and gratitude towards all previous organisers are passed on from congress to congress by each previous main organiser becoming a member of the next programme and organising committee, as proposed at the very beginning. Throughout the centuries, Central Europe has always been a very influential melting pot of ideas for transnational and regional cooperation. At the end of the last century and the beginning of this century, there was a great spirit of cooperation in Central Europe, as first evidenced by the Alps-Adriatic Working Community and later by the Danube Initiatives – these two initiatives had a wide variety of cross-border motives for cooperation, not only political alliances. Obviously, various platforms had asserted a necessity for cooperation. However, the Central European initiative (CEI), established in Budapest in 1989, remains the one that provides a good framework for scientific research and educational cooperation in the area of food. The expansion of the European Union to Central Europe later stimulated a new visionary cooperation, not only in the North–South orientation, but also in the West–East orientation. This was the milestone for the establishment of the Central European Congress on Food and Nutrition (CEFood congress) in 2002 in Ljubljana.

CEFood 2022 offered an excellent scientific programme with the best speakers from the Central European initiative countries. In addition, the congress offered other experiences that highlighted Slovenian cuisine and its gastronomic heritage. Therefore, the participants were able to identify and appreciate the latest advances along the food supply chain, including research and education with open discussions on current issues in this field. Environmental concerns were considered, guidance to the ongoing projects in food science, technology and nutrition was offered, and hands-on professionals, scholars, researchers and policy makers were involved.

The outcome of the intensive work of the organisers are two publications: *CEFood Congress Book* with 336 pages and *Food, nutrition and environment: Positions in Central European space* with 176 pages. In 100 hours of scientific programme, participants enjoyed 8 plenary lectures, 11 keynote lectures and 34 oral presentations in 11 sessions, 5 flash presentations, 144 posters and 13 project presentations at the Central European Project Day, 11 contributions at the Central European Inspiration Food Day. The event was attended by 249 participants from 27 countries, including 233 from the CEI countries. The support by 12 sponsors provided 17 grants for students from economically disadvantaged countries.

The scientific programme of the pre-congress event, the Central European Food Project Day, focused on the presentation of food-related R&D projects in the fields of food security, food technology and nutrition, and food safety. The main programme sessions covered the following topics: Food, nature and health; From technology to food; Before and after the harvest; Plants ‐ a staple food or an alternative?; Potentials and limitations of bioactive compounds; New food products, technologies and techniques; Can we improve food properties and prevent food fraud at the same time?; Food related health hazards and risks; Nutrition: is the point in the food or in the gut?; Biotechnology: evolution or revolution?; Food microbes: friends or foes?; Consumer’s food choices and risk management; and Food analytics. All scientific sessions were dedicated to Slovenian food professionals or scientists who have contributed in the past to the development and breakthroughs in the areas of science.

The latest achievements in science and good practices were also shared in the World Cafe on food supplements, at the round table ’Edible insects ‐ food of the future?’ and in three workshops: ’Challenges for beer and wine in today’s food world’, ’Food and its safety in a fast changing world’ and ’Modern food: local vs. global, traditional vs. innovative in the “healthy” perspective’. In addition, the Central European Inspiration Food Day (CE-I-FooDay) offered younger professionals from each CEI country the opportunity to write a position paper, which was published in a special publication, and presented orally at CE-I-FooDay as an event after the CEFood 2022 Congress.

Considering the current world and food situation, the CEFood congress provided an important contribution to new concepts and views of individual scientists/specialists/teachers in the field of food, technology and nutrition as a scientific advocate for healthy people in a healthy environment, in the future society from a food perspective. Concerning this special issue, a very rigorous selection from among sixteen submitted papers has resulted in the content in front of you - it brings a comprehensive review paper, two original scientific papers and a preliminary communication. The review article focuses on the application of minimal processing technologies for the production and preservation of tailor-made foods – with a range of approaches, including traditional and emerging technologies, and novel ingredients such as biomolecules from diverse sources, including microorganisms. Combined approaches based on the principles of hurdle technology produce effective synergistic effects that improve food safety and shelf-life extension of tailor-made foods while preserving their functional properties. The included scientific papers all target new products with improved functional characteristics – from the bakery raw materials, such as whole wheat flour with improved digestibility and antioxidant properties due to the effect of ultrasound and hydrothermal treatment, to the final goods with new functional ingredients, such as muffins with chia and lyophilised peach powder, and almond-based dairy-free milk alternative formulation fortified with plant extracts – all with the optimised quality attributes to meet the expectations of the contemporary consumer.

We, the Guest Editors of this special issue, would like to thank the Editor-in-Chief of the journal, Professor Vladimir Mrša, for providing the valuable space in the journal to accommodate the selected contributions, to Zrinka Pongrac Habdija and Iva Grabarić Andonovski and the production team of FTB for their great support in completing this special issue. Our thanks also go to the reviewers for their insightful comments and for improving the quality of the contributions included in this issue. We would also like to thank all the authors of this special issue for their cooperation in its publication.

Guest Editors

Peter Raspor

Sonja Smole Možina

Irena Vovk

